# The role of mitochondrial DNA mutations and free radicals in disease and ageing

**DOI:** 10.1111/joim.12055

**Published:** 2013-03-07

**Authors:** M Lagouge, N-G Larsson

**Affiliations:** Department of Mitochondrial Biology, Max Planck Institute for Biology of AgeingCologne, Germany

**Keywords:** ageing, antioxidants, mtDNA, ROS

## Abstract

Considerable efforts have been made to understand the role of oxidative stress in age-related diseases and ageing. The mitochondrial free radical theory of ageing, which proposes that damage to mitochondrial DNA (mtDNA) and other macromolecules caused by the production of reactive oxygen species (ROS) during cellular respiration drives ageing, has for a long time been the central hypothesis in the field. However, in contrast with this theory, evidence from an increasing number of experimental studies has suggested that mtDNA mutations may be generated by replication errors rather than by accumulated oxidative damage. Furthermore, interventions to modulate ROS levels in humans and animal models have not produced consistent results in terms of delaying disease progression and extending lifespan. A number of recent experimental findings strongly question the mitochondrial free radical theory of ageing, leading to the emergence of new theories of how age-associated mitochondrial dysfunction may lead to ageing. These new hypotheses are mainly based on the underlying notion that, despite their deleterious role, ROS are essential signalling molecules that mediate stress responses in general and the stress response to age-dependent damage in particular. This novel view of ROS roles has a clear impact on the interpretation of studies in which antioxidants have been used to treat human age-related diseases commonly linked to oxidative stress.

## Overview of the evolution of the mitochondrial theories of ageing

Common human diseases, such as atherosclerosis, diabetes mellitus, cancer and various forms of neurodegeneration, are often associated with age. Thus, ageing is a main risk factor for many diseases and an understanding of the molecular mechanisms driving the ageing process may therefore lead to new insights into the pathophysiology of such diseases. Why and how do we age? The causes of normal ageing are likely to be multifactorial with no single mechanism able to explain all aspects. A detailed analysis of the numerous theories of ageing is beyond the scope of this review and here we will focus on the role of mitochondrial DNA (mtDNA) mutations and reactive oxygen species (ROS) in ageing. We will describe the basic principles of the mitochondrial theory of ageing and how it has evolved over the past decades. New concepts have recently emerged concerning the role of ROS in cellular function and ageing. We will discuss the possible reasons why intervention studies to reduce ROS levels in both humans and animal models have generated different and often inconclusive results concerning the role of oxidative stress in age-dependent human pathologies.

### The free radical theory of ageing

In the 1950s, Harman proposed the free radical theory of ageing which postulated that the production of intracellular ROS, and its deleterious effects on various cellular components, was the major determinant of lifespan 1. This landmark theory initiated the molecular era of ageing research. With the discovery of the superoxide dismutase (SOD) enzymes in the late 1960s 2, it became evident that specific enzymatic scavenging systems have evolved to detoxify superoxide and other types of ROS.

### The mitochondrial free radical theory of ageing

The mitochondrial respiratory chain (RC) is a major site of production of ROS in the cell and it has therefore been suggested that mitochondria are the prime targets for oxidative damage. Recognition of the important role of mitochondria led to a refinement of the free radical theory by Harman and others 3, 4 into the mitochondrial free radical theory of ageing, which considers mtDNA mutations to be the initiating, primary event in the ageing process.

**Fig. 1:**
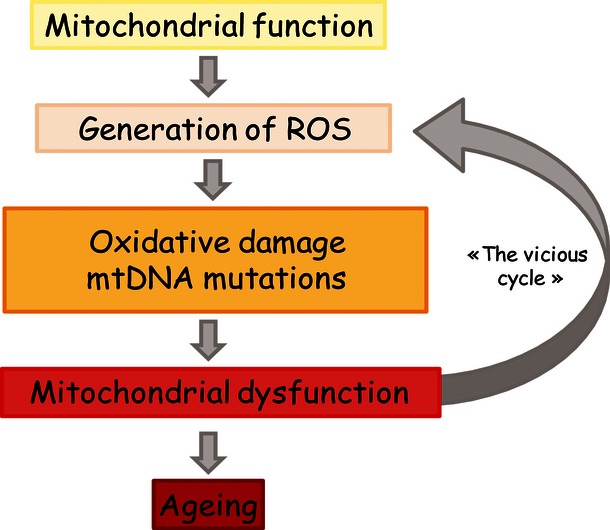
Schematic representation of the mitochondrial free radical theory of ageing. Reactive oxygen species (ROS) are normal by-products of mitochondrial function that progressively damage the constituents of mitochondria, inducing mitochondrial dysfunction and increased ROS production through a vicious cycle, leading ultimately to cellular dysfunction and ageing.

The mitochondrial free radical theory (Fig. 1) postulates that ageing is caused by the toxicity of ROS, initiating a vicious cycle whereby damage to mtDNA and other mitochondrial constituents leads to RC dysfunction, which in turn leads to increased generation of ROS further facilitating mtDNA damage and thus creating a self-amplifying deterioration. This appealing theory provides a very interesting conceptual framework and has stimulated many crucial discoveries in the fields of mitochondrial function and ageing. However, this model has also been extensively debated as the links between the postulated underlying cornerstone mechanisms are rather correlative and as there is a lack of conclusive experimental evidence to support this theory. It is important to note that there have also been interesting attempts to unify this theory with other schemes, whereby age-related changes in the nucleus (e.g. telomere dysfunction) trigger mitochondrial dysfunction 5.

### The gradual ROS response hypothesis

The mitochondrial free radical theory of ageing postulates that ROS are damaging and that the resulting damage drives ageing. However, new theories have emerged in which ROS are predicted to have more subtle roles. Over the past decade, it has been progressively appreciated that ROS can function as signalling molecules, facilitating adaptation to stress in a wide variety of physiological situations 6. In line with this, Hekimi and colleagues 7 have formulated an interesting hypothesis in which ROS generation is not a cause of ageing, but rather represents a stress signal in response to age-dependent damage. This model will be discussed further in this review, as it provides a new way of interpreting experimental results that were previously conceptually conflicting with the mitochondrial free radical theory of ageing.

## The impact of mtDNA mutations on mitochondrial function and ageing

**Fig. 2:**
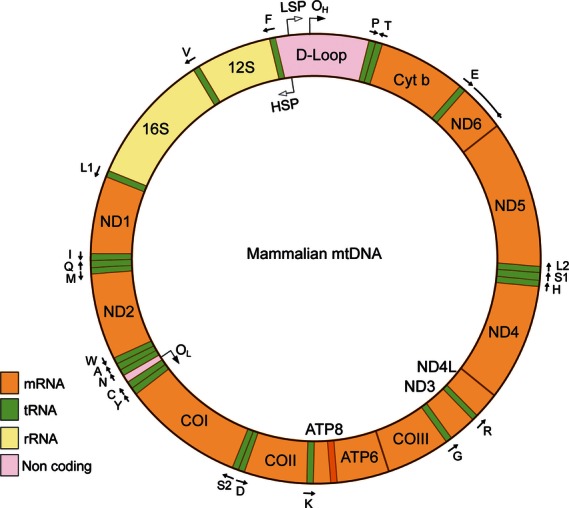
Organization of the mammalian mtDNA. The mammalian mitochondrial DNA (mtDNA) is a double-stranded circular molecule. The two strands are denoted the heavy (H) and the light (L) strands. The noncoding D-loop region contains the promoters for the H and L strands (HSP and LSP) as well as the origin of replication of the leading strand of mtDNA (O_H_). Transcription from HSP produces two rRNAs (12S and 16S rRNA), 10 mRNAs (the monocistronic ND1–3, ND5, Cyt b and COI–III mRNAs, and the biscistronic ND4/ND4L and ATP6/ATP8 mRNAS) and 14 tRNAs (F, V, L1, I, M, W, D, K, G, R, H, S1, L2 and T). Transcription from LSP produces one mRNA (ND6) and eight tRNAs (P, E, S2, Y, C, N, A and Q). *Reproduced from* 14.

Mitochondria are the only organelles, besides the nucleus, which contain their own DNA. Human mtDNA is a 16.6 kb circular, double-stranded molecule, encoding 11 mRNAs that give rise to 13 subunits of the oxidative phosphorylation system, 22 transfer RNAs (tRNAs) and two ribosomal RNAs (rRNAs) that are essential for mitochondrial translation (Fig. 2). The rest of the ∼10^3^ mitochondrial proteins are encoded by the nuclear genome and imported into the mitochondria 8.

### Genetics of mtDNA

**Fig. 3:**
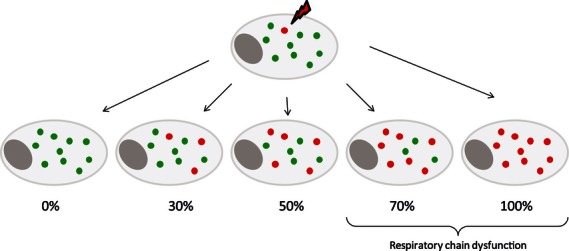
Mitotic segregation of mtDNA. A single mutational event within a mitochondrial DNA (mtDNA) molecule creates heteroplasmy in a cell. Mutated mtDNA molecules are shown in red and normal mtDNA molecules in green. There is no synchronization between mtDNA replication and cell division, which means that a particular mtDNA molecule, mutated or not, may be replicated many times or not at all during a single cell cycle. Repeated cell division will lead to mitotic segregation of normal and mutated mtDNA molecules. Accumulation of mutated mtDNA above a certain threshold level will lead to impaired respiratory chain function.

Mitochondrial DNA is exclusively maternally transmitted in mammals, and the oocyte contains ∼10^5^ copies. The mtDNA copy number in a somatic mammalian cell is considerably lower, with ∼10^3^–10^4^ copies of mtDNA per cell. A mutation arising in a single mtDNA molecule can expand clonally during mtDNA replication in somatic cells, because there is no mechanism to ensure that every mtDNA molecule is replicated only once in coordination with replication of nuclear DNA during each cell cycle. This so-called somatic or mitotic segregation leads to random segregation of mtDNA mutations as the cell divides 9 and can result in daughter cells that exhibit homoplasmy (i.e. that only contain normal or mutated mtDNA) or heteroplasmy (i.e. that contain a mixture of normal and mutated mtDNA molecules). The principle of mitotic segregation explains how a cell carrying low levels of a mutated mtDNA molecule can give rise to daughter cells with high mutation levels (reviewed in 10) (Fig. 3). This type of mitotic segregation has been observed both for point mutations and large deletions of mtDNA. However, there is also some evidence that apparently neutral polymorphisms may segregate in a nonrandom fashion and that selection can be tissue-specific 11.

A large number of mtDNA mutations and deletions have been associated with various clinical phenotypes (for review see 12). A minimal threshold level of a pathogenic mtDNA mutation must be present in a cell to cause RC deficiency. Different types of heteroplasmic mtDNA mutations often have different thresholds for the induction of RC deficiency, ranging from 90% for some tRNA mutations to 60% for large mtDNA deletions (Fig. 3). Patients carrying heteroplasmic mtDNA mutations often have widely varying levels of mutated mtDNA in different organs and even in different cells of a given organ 8. The phenotype caused by each of these mutations will be determined by the ratio of mutant to normal mtDNA molecules within the affected tissue, which in turn determines the proportion of cells that will be RC-deficient. Mouse chimeras with mosaic RC deficiency in pyramidal neurons of the neocortex developed clinical symptoms if the proportion of RC-deficient neurons was >20%, and the defect caused mortality if the proportion of defective neurons reached >60–80% 13. This finding provides experimental evidence that the proportion of RC-deficient cells in a given tissue determines the clinical severity and outcome of the disease.

### Accumulation of mtDNA mutations with age

It has been observed that mutated mtDNA molecules accumulate with age. Methods for quantification of mtDNA point mutations and deletions are not straightforward; for further discussion of this topic, see our recent review 14. The age-dependent accumulation of mutations in mtDNA can in principle be explained by two main mechanisms, replication errors and unrepaired damage. First, it has been suggested that the massive mtDNA replication occurring during embryogenesis will result in replication errors due to the inherent error rate of the mtDNA polymerase 14. The mtDNA mutations formed during embryogenesis will be subjected to segregation and clonal expansion in postnatal life. Secondly, it has been alternatively proposed that accumulated damage, for example caused by ROS, will overwhelm the repair machinery and result in accumulation of mtDNA mutations. According to the mitochondrial free radical theory of ageing (Fig. 1), damage accumulation will create a vicious cycle resulting in an exponential increase in mtDNA mutations with time.

There are mechanisms to maintain mtDNA integrity, but the number of available repair systems seems to be much more limited in mitochondria than in the nucleus. It should be emphasized that mtDNA is not naked but rather packaged into protein – mtDNA aggregates termed nucleoids 15. These nucleoids have an average diameter of 100 nm and frequently contain a single copy of mtDNA. This compaction is likely to physically protect mtDNA from chemical damage. Base excision repair (BER) is the main repair mechanism in mammalian mitochondria 16 and is present in its short- and long-patch variants. There are also some indications that double-strand break repair and other direct repair mechanisms may be present in mammalian mitochondria, but these other repair mechanisms still need to be molecularly defined. For an extensive description of different mtDNA repair mechanisms, see review by Boesch and colleagues 17. The mechanisms that impair or restrict the efficiency of the repair machinery with ageing remain unclear, but a decline in the mitochondrial import capacity with age could be one such mechanism. For instance, it is considered that the 8-oxo-2′deoxyguanosine (8-oxoG) is one of the most abundant oxidative lesions that accumulates in mtDNA over time. This lesion is known to induce transversion mutations due to the mispairing of 8-oxoG with adenine during replication. Of interest, it has been reported that accumulation of 8-oxoG in mtDNA occurs with age possibly because OGG1, a DNA glycosylase enzyme involved in BER of 8-oxoG, accumulates in a unprocessed form in the mitochondrial intermembrane space and fails to be imported inside the matrix 18. The 8-oxoG adduct, if unrepaired, will result in G to T transversion mutations. In a recent deep sequencing study, we found no evidence that mtDNA transversion mutations increased with age in the mouse, thus suggesting that this type of damage may not be a major source of formation of mtDNA mutations and that replication errors may have a more important role 19.

### The causative role of mtDNA mutations in ageing

Mitochondrial DNA mutator mice were generated about 10 years ago 20, 21 to experimentally determine the impact of mtDNA mutations on mitochondrial function and ageing. mtDNA mutator mice have a severe defect in the proofreading function of the mtDNA polymerase PolG, leading to the progressive and random accumulation of mtDNA point mutations in the course of mitochondrial biogenesis. As proofreading in these mice is much reduced, they develop a mtDNA mutator phenotype with a 3- to 5-fold increase in the level of point mutations, as well as an increased number of linear deleted mtDNA molecules (reviewed in 10). These mice exhibit premature ageing and a reduced lifespan 20 and therefore provide experimental evidence of a causative link between mtDNA mutations and induction of ageing phenotypes in mammals.

Despite the great advances in understanding the association between mtDNA mutations and ageing provided by the mtDNA mutator mouse model, some mechanistic aspects remain to be clarified. First, mtDNA mutator mice show a rapid accumulation of mtDNA mutations during embryogenesis and thereafter a more linear increase in the mtDNA mutation burden in adult animals. The kinetics of accumulation of mutations in these mtDNA mutator mice is not compatible with the mitochondrial free radical theory of ageing, as this theory predicts an exponential increase in the mutation burden throughout life. The mtDNA mutator mouse certainly shows that high levels of mtDNA mutations can cause a premature ageing syndrome, but this finding does not prove that the levels of mtDNA mutations seen in normal ageing are sufficiently high to cause ageing-related pathology. A critical experiment, yet to be conducted, is to determine whether a reduction in somatic levels of mtDNA mutations will prolong lifespan in an experimental model.

Recent additional characterization of the mtDNA mutator mouse 22 has shown that it has a stem cell phenotype that may explain at least in part the ageing phenotypes. Neural stem cells (NSC) showed decreased renewal *in vitro* and quiescent pools of NSC were decreased, whereas the haematopoietic stem cells showed a skewed lineage differentiation leading to anaemia and lymphopenia 22. The mtDNA deletor mice generated by Tyynismaa *et al*. 23 accumulate large-scale mtDNA deletions in postmitotic tissues and exhibit late-onset RC deficiency, similar to the mutator mice. However, the deletor mice show no signs of premature ageing and, in contrast to the mtDNA mutator mice, have no obvious somatic stem cell phenotypes 22 (Table 1). These observations support a crucial role of somatic stem cell dysfunction in generating the progeroid phenotype seen in mtDNA mutator mice.

**Table 1:** Comparison between the main features of the mtDNA mutator and deletor mouse model phenotypes

	mtDNA	mtDNA
	mutator mice	deletor mice
Genetic intervention	Mutated PolgA	Mutated Twinkle
Mutation type	Point mutations + linear mtDNA with deletions	Circular mtDNA with deletions
Respiratory chain deficiency	Late onset	Late onset
Somatic stem cells	Dysfunction	No phenotype
Ageing	Premature ageing	Normal lifespan

mtDNA, mitochondrial DNA.

Deletor mice show no signs of premature ageing and, in contrast to mtDNA mutator mice, have no obvious somatic stem cell phenotypes. This supports a crucial role for somatic stem cell dysfunction in generating the progeroid phenotype seen in mtDNA mutator mice.

## Role of ROS and antioxidant defence in mitochondrial function and ageing

### ROS, antioxidants and oxidative damage

**Fig. 4:**
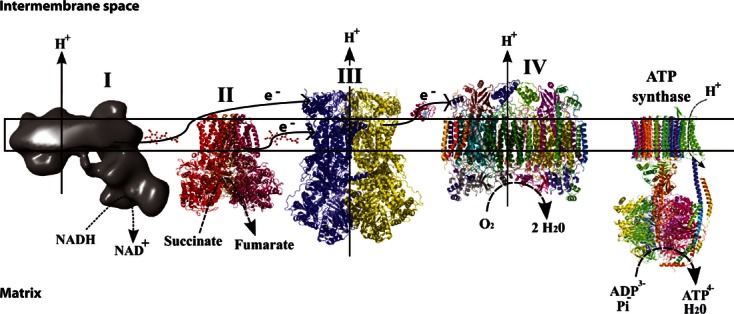
The oxidative phosphorylation system. The respiratory chain, composed of four complexes (I to IV), transfers electrons (e-) in a stepwise manner until they finally reduce O_2_ to form H_2_O. Electron transfer is coupled to a vectorial proton translocation outside the matrix space through three of the four complexes (I, III and IV). Protons accumulate and create an electrochemical gradient across the inner mitochondrial membrane. This osmotic energy is used to drive ATP synthesis as the protons re-enter the mitochondrial matrix through ATP synthase (the complexes are not drawn to scale). *Adapted from* 109.

Reactive oxygen species are generated in multiple cellular compartments, but the vast majority (about 90%) of cellular ROS can be traced back to the mitochondria 24. The generation of mitochondrial ROS is a normal consequence of oxidative phosphorylation, an essential process of the mitochondrial network, which couples the oxidation of reduced nicotinamide adenine dinucleotide (NADH) or succinate by the RC to ATP synthesis. The substrates NADH and succinate deliver electrons to the RC, which is composed of four complexes (I to IV) that transfer electrons in a stepwise manner to finally reduce O_2_ to form water. Three of the four complexes (I, III and IV) couple the electron transfer to vectorial proton translocation outside the matrix space. Due to the low proton conductance of the inner mitochondrial membrane, protons accumulate and create an electrochemical gradient across the inner mitochondrial membrane. This osmotic energy is used to drive ATP synthesis as the protons re-enter the mitochondrial matrix through ATP synthase (25; reviewed in 24) (Fig. 4).

**Fig. 5:**
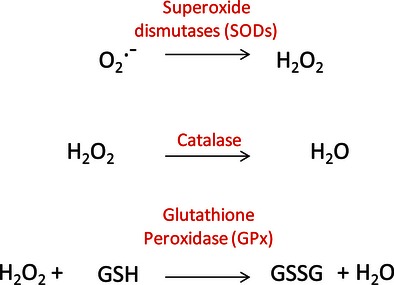
Reactive oxygen species (ROS) detoxification enzymes. The cell is equipped with a variety of defence mechanisms to scavenge ROS. Superoxide dismutases catalyse the dismutation of superoxide into oxygen and hydrogen peroxide. Catalase reacts with the hydrogen peroxide to catalyse the formation of water and oxygen. Glutathione peroxidase reduces hydrogen peroxide. GSH stands for reduced glutathione whereas GSSG stands for oxidized glutathione.

At several sites along the RC, electrons derived from NADH or succinate oxidation may escape and react with O_2_ or other electron acceptors to generate free radicals. Complexes I and III are predicted to be the major sites for ROS production 26. The reduction of O_2_ by one electron generates the superoxide anion (O_2_·^**−**^), which can then be further reduced to the hydroxyl radical (OH·^**−**^) and hydrogen peroxide (H_2_O_2_) 27. These ROS can damage lipids, proteins and DNA (e.g. create the 8-oxoG lesion described above). Of note, the cell is equipped with a variety of defence mechanisms to scavenge ROS. Superoxide is converted to H_2_O_2_ by two intracellular SODs, Cu–Zn SOD (SOD1) and MnSOD (SOD2); H_2_O_2_ can then be transformed into H_2_O by catalase or glutathione peroxidase (GPx) (Fig. 5). The cell also contains nonenzymatic ROS scavengers such as ascorbate, flavonoids, carotenoids and glutathione, which may all contribute to the inactivation of otherwise damaging ROS (reviewed in 10). Thus, oxidative stress results from an imbalance between ROS generation and detoxification and thereby creates oxidative damage. It is also important to note that oxidative damage, once generated, may be removed by repair mechanisms. Increased levels of oxidative damage to macromolecules may therefore be explained either by increased ROS formation or by decreased repair of ROS-induced adducts.

It is important to note that oxidative stress has been reported to be involved in numerous cellular processes but unfortunately, the supporting experimental data are not straightforward to interpret. As discussed in the previous paragraph, ROS production is important but the ROS-scavenging systems as well as repair mechanisms for oxidative damage should also be considered to evaluate the net outcome of oxidative stress 28, 29. In addition, the performance of different methods used to directly measure levels of different ROS types is heavily debated 29. Because of discrepancies in the methods used, the conclusions of some studies attributing a role to ROS are questionable. For example, the mitochondrial content of protein oxidation products, such as nitrotyrosine and protein carbonyls, is often used as a marker of mitochondrial oxidative stress. However, an increased content of protein carbonyls and protein nitration adducts does not necessarily reflect increased oxidative stress, but can also be the result of a reduced turnover of damaged proteins 29. In line with this, assays to determine the activity of antioxidant enzymes (e.g. SOD2 and GPx) and DNA repair enzymes (e.g. 8-oxoguanine DNA glycosylase) are often used to assess ROS production. Decreased activity of ROS scavenging and DNA repair enzymes has been reported to cause increased oxidative stress but, paradoxically, increased activity of these enzymes is typically interpreted as a sign of increased oxidative stress 30. In addition, experiments to measure levels of ROS in biological systems are often conducted with redox-sensitive probes, which can be highly susceptible to other chemical reactions not dependent on ROS levels. For instance, there are many problems with respect to the specificity of the oxidation of dichlorodihydrofluorescein (DCFH) resulting in production of fluorescent dichlorofluorescein (DCF), which is often used as a probe to measure ROS, in particular H_2_O_2_ production 31. Caveats also apply to the interpretation of results obtained with other probes such as MitoSOX and Amplex Red, which also have side reactions and a range of associated artefacts 32, 33. As stated by Murphy and colleagues 29 there is an urgent need to precisely define valid protocols to measure ROS and to develop better and more specific probes to measure the different types of ROS. Considerable progress has been made in the development of probes for live imaging, exemplified by the redox-sensitive fluorescent probes roGFP 34 and HyPER 35, 36, which react selectively with particular ROS and then undergo changes in fluorescence with a high degree of sensitivity. Recently, Albrecht and colleagues 37 demonstrated the efficacy of two different genetically encoded redox probes in the measurement of glutathione redox state and H_2_O_2_ levels *in vivo* in the tissues of *Drosophila melanogaster*. Similarly, Cochemé and co-workers created the MitoB probe that reacts with H_2_O_2_ to form the phenol MitoP. Quantification of the MitoP/MitoB ratio using a mass spectrometry approach enabled the *in vivo* measurement of mitochondrial H_2_O_2_ levels in thoracic muscles of *D. melanogaster* 38. Hopefully, these and future technical advances will help to unravel the exact role of ROS and oxidative damage in the ageing process in a more sensitive and accurate way *in vivo*.

### The causative role of ROS in the ageing process

The involvement of ROS in the creation of mtDNA mutations is central to the mitochondrial free radical theory of ageing. Numerous descriptive studies have demonstrated an age-related increase in oxidative damage to lipids, proteins and DNA, but interventions to lower ROS have not produced consistent results in terms of lifespan extension (Table 2). Thus, evidence is conflicting, providing arguments both for and against the mitochondrial free radical theory of ageing.

**Table 2:** Summary of *in vivo* genetic interventions to modulate ROS detoxification capacity and their effects on lifespan

Model organism	*Caenorhabditis elegans*	*Drosophila melanogaster*		*Mus musculus*					
Targeted enzyme	SOD2 ↓	SODs ↓	SODs ↑	Catalase ↑	SODs Catalase ↑	SOD2 ↓	SOD2 GPx1 ↓	SOD2 ↓	GPx4 ↓
ROS/Oxidative damage	↑ ∼	↑	↑	↓	↓	↑	↑	n.d.	↑
Lifespan	∼	↓	∼	↑	∼	∼	∼	↓	↑
References	53–56	39, 40	51	38	43	44	45	41, 42	46

ROS, reactive oxygen species; SOD, superoxide dismutase; GPx, glutathione peroxidase; n.d., not determined.

Genetic interventions to modulate ROS levels do not produce consistent results in terms of lifespan extension. ↑, increase; ↓, decrease; ˜ no change.

It has been shown that mice overexpressing the catalase enzyme targeted to the mitochondria exhibit an increase in lifespan of 5 months 39, but this finding should be interpreted with caution because catalase is normally not present in mitochondria. In line with these results, RNA interference-mediated silencing of *Sod2* in *D. melanogaster* correlates with early adult-onset mortality 40, as does a null mutation for the same gene 41. Similarly, *Sod2* knockout mice exhibit neonatal lethality 42, 43. These results support the free radical theory of ageing and reinforce the proposal that mitochondria are a source of these radicals.

By contrast, work by Perez and colleagues showed that overexpression of single or different combinations of antioxidant enzymes, for example, SOD1, SOD2 and catalase, decreases oxidative damage without extending lifespan in transgenic mice 44. In line with this observation, mice with a mild reduction in SOD2-dependent antioxidant defence (*Sod2* heterozygous knockout mice) did not show an accelerated ageing phenotype or shorter lifespan 45 despite exhibiting higher levels of oxidative stress in comparison with wild-type littermates. Similarly, mice with combined partial deficiencies of SOD2 and GPx1 have increased oxidative damage but no reduction in longevity compared with controls 46. Even more striking is the fact that, despite higher levels of oxidative damage biomarkers, median lifespan is increased in mice with partial inactivation of GPx4 (*Gpx4* heterozygous knockout mice) 47. As already mentioned, mtDNA mutator mice accumulate mtDNA mutations in a linear manner in adult life 20 and have no major increase in oxidative damage in many different tissues 48; these findings do not support the mitochondrial free radical theory of ageing. These experimental results have led to the conclusion that the premature ageing syndrome in mtDNA mutator mice is not caused by a vicious cycle of oxidative stress and exponential accumulation of mtDNA mutations. Interestingly, it has recently been suggested that *Tfam* heterozygous knockout mice, which undergo mild mtDNA depletion 49, also exhibit increased oxidative mtDNA damage susceptibility although the underlying mechanism is unclear 50; therefore, these mice may represent a useful model to assess the role of oxidative damage in ageing. By contrast, in cardiomyocytes from *Tfam* homozygous knockout mice, which undergo mtDNA depletion associated with severe RC dysfunction, the initial increased ROS production is neutralized by induction of the antioxidant defences, as illustrated by the measurement of GPx and aconitase enzyme activities 51. Studies in fruit flies, similar to those in mice, have shown that overexpression of antioxidant enzymes does not necessarily extend lifespan 52. Consistent with the results from these genetic studies, pharmacological intervention with antioxidants also failed to promote longevity in the fruit fly 53. *Caenorhabditis elegans* has two isoforms of the mitochondrial SOD (encoded by *Sod2* and *Sod3*) and deletion of neither *Sod2* nor *Sod3* decreases survival. Even *Sod2*/*Sod3* double mutants, entirely deficient in mitochondrial SODs, show no reduction in lifespan 54–56. Of interest, this result cannot be explained by a compensatory effect due to the three other SODs present in *C*. *elegans* as it has recently been shown that animals completely lacking SOD activity (sod-12345 worms) have normal levels of oxidative stress and a normal lifespan 57. In conclusion, there is now an abundance of experimental evidence that does not support the mitochondrial free radical theory of ageing despite much data showing correlations between ROS and ageing.

Dietary restriction (DR), also referred to as caloric restriction, is the most widely used experimental manipulation known to increase lifespan and delay ageing in a variety of organisms. Results from experimental DR were initially thought to support the mitochondrial free radical theory of ageing as DR, by reducing mitochondrial activity, decreases ROS production and thereby reduces accumulation of oxidative damage 58–60. However, it has also been shown that DR actually induces a transient increase in ROS production (reviewed in 61). Schulz and colleagues reported that reduced glucose availability in *C*. *elegans* promotes the formation of ROS, induces catalase enzyme activity and increases oxidative stress resistance and survival 62. A possible explanation is that DR transiently induces increased ROS production, which leads to enhanced stress defence 62, which in turn results in a reduction of the net stress level 63. This model may explain the positive effects of DR on longevity and may also explain why antioxidants often fail to promote longevity, as they supposedly interfere with defence pathways 61. According to the DR model, ROS may serve as a molecular signal to induce endogenous defence mechanisms to promote stress resistance and longevity. This adaptive response to a potentially harmful molecule has been termed mitohormesis 64.

In mammals, there are seven sirtuins (SIRT1–7) that are orthologues of the yeast silent information regulator (*Sir2*) gene 65. They have an extremely wide range of biological functions, exerted through their ability to catalyse NAD^+^-dependent deacetylation, desuccinylation or ADP-ribosylation of their numerous substrates (reviewed in 65, 66). The seven mammalian sirtuins have distinct subcellular localizations, and it has been reported that SIRT3, SIRT4 and SIRT5 are present within mitochondria 67, 68. SIRT3 expression can be induced by ROS 69 and DR 69, 70. Under DR, SIRT3 has been shown to deacetylate and subsequently activate SOD2 69, 71, leading to reduced oxidative stress. Of note, the protective effects of DR on oxidative stress and damage are reduced in mice lacking SIRT3 71. Beyond the activation of SOD2, SIRT3 also seems to have other antioxidant functions in mitochondria. For instance, a comparative study of *Sirt3* wild-type and knockout mice has shown that SIRT3 mediates the beneficial effects of DR on age-related hearing loss and prevents the accumulation of ROS under DR in cochlea cells through the deacetylation and activation of isocitrate dehydrogenase 2 (IDH2) 70. In the context of DR, SIRT3 also activates glutamate dehydrogenase (GDH) (reviewed in 72). Activation of IDH2 and GDH indirectly stimulates the production of reduced glutathione, the cofactor used by glutathione peroxidase (GPx) to detoxify ROS (reviewed in 72, 73). SIRT3 is the only sirtuin for which there is some genetic evidence of a relationship with human ageing 74 and it is important to note that the direct involvement of the sirtuins in the longevity process has recently been questioned in invertebrates 75 as well as in mammals 76. Nevertheless, the remarkable fact that SIRT3, the activity of which is induced by DR and/or ROS sensing, seems to be able to directly reduce ROS levels supports the proposals that: (i) DR indeed induces increased stress resistance, and (ii) induction of SIRT3 expression is a DR-induced defence mechanism.

### ROS as signalling molecules: the gradual ROS response theory of ageing

**Fig. 6:**
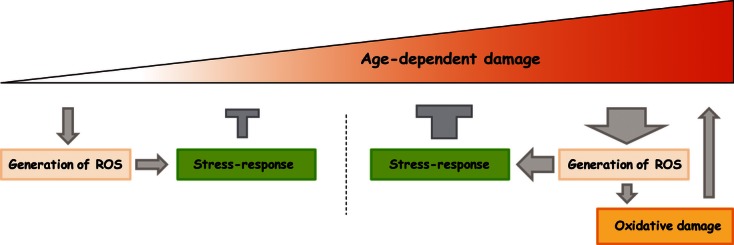
The gradual reactive oxygen species (ROS) response hypothesis. Reactive oxygen species-independent age-related damage induces the production of ROS as part of a stress-response defence pathway (left). At this stage, toxicity is well controlled by the antioxidant system and is therefore not deleterious. ROS-independent damage accumulates with age and induces a progressive increase in ROS generation as the cell attempts to increase its stress response (right). The antioxidant system is overwhelmed by the increased amount of ROS, and ROS-induced damage will occur and contribute to the total increase in age-associated damage (right). ROS thus have a dual role in ageing: initially ROS promote signalling to prevent damage accumulation; later, ROS contribute to damage creation. *Adapted from* 7.

The mitochondrial free radical theory of ageing is based on numerous observations, including: (i) age is correlated with increased levels of oxidative damage, (ii) oxidative phosphorylation capacity decreases with ageing, and (iii) several age-associated diseases are associated with increased oxidative stress, as well as the (questionable) prediction that mitochondrial dysfunction should increase ROS production. This theory, based on correlations, has provided a very interesting framework and has led to interventions aimed at decreasing the level of ROS to generate health benefits. However, several lines of evidence reported here and elsewhere 7, 30, 61, 77 are incompatible with the mitochondrial free radical theory of ageing. Experimental results suggest that the increase in ROS is a consequence rather than a cause of ageing 7 and that ROS are associated with ageing in the sense that they are mediators of the stress response to age-dependent damage. According to this theory, ROS generation increases gradually with age until it reaches a level at which toxicity starts to generate the damage that it was meant to alleviate 7 (Fig. 6). In this model, which shares some common features with the mitohormesis, ROS are not only deleterious agents, but also important signalling molecules. The finding that ROS may serve as a molecular signal to induce endogenous defences is not new and has been observed in settings other that DR. ROS have indeed been described as signalling molecules facilitating cellular adaptation to stress in a wide variety of physiological situations. For instance, they have been shown to regulate adaptation to hypoxia, autophagy, immune cell function and cellular differentiation 6. The concept of ROS as signalling molecules is now proposed to allow adaptation to ROS-independent age-dependent damage in a more general way. Additional support for the gradual ROS response hypothesis of ageing comes from Lapointe and colleagues, who showed that increased oxidative stress in mitochondria of young *Mclk1*^+/−^ mice protects against age-dependent loss of mitochondrial function 78. The neural and haematopoietic progenitors from mtDNA mutator mice exhibit impaired function from embryogenesis onwards, and this stem cell dysfunction can be rescued by treatment with the antioxidant cysteine precursor N-acetyl-L-cysteine throughout pregnancy 22. To a certain extent, these findings 22 support the gradual ROS response theory by Hekimi and co-workers as they suggest that (i) a subtle increase in mitochondrial ROS, as a consequence of mtDNA mutagenesis, is sufficient to modify the signalling in somatic stem cells and to disrupt their homoeostasis, and (ii) premature ageing may be accounted for by a cell type – specific ROS-signalling mechanism and not by a general increase in ROS damage and subsequent RC dysfunction.

There are some indications that soluble hormone-like factors, termed ‘mitokines’, can be produced in a limited number of cells in response to mitochondrial stress and influence distant tissues with normal RC function 79. Experimental work in mice has shown that fibroblast growth factor 21 (FGF21) fulfils many of the criteria of a mitokine as it is produced by skeletal muscle in response to mitochondrial stress and is able to signal to distant tissues. As described previously, the deletor mouse model exhibits a progressive accumulation of large-scale mtDNA deletions in brain and muscle, associated with late-onset RC deficiency 23. A detailed analysis of the muscle fibres of these animals shows that the RC deficiency induces a local starvation-like stress response associated with FGF21 secretion, which can influence signalling in other tissues 80. Indeed, the expression of FGF21 is increased in the muscles of deletor mice, via activation of the Akt1 signalling pathway, but not in tissues in which it is commonly induced upon fasting 81, 82. This tissue-specific expression correlates with a global increase in FGF21 levels in plasma. Very interestingly, FGF21 expression follows exactly the progression of the RC deficiency as evaluated by the number of cytochrome c oxidase (COX)-negative muscle fibres 80. Moreover, it has been suggested that RC dysfunction may be the cause of the induction of FGF21 expression in muscle; this is supported by the observations that (i) the classic inducers of FGF21 such as fasting and exercise fail to induce FGF21 expression in healthy muscles, and (ii) FGF21 expression was induced in another model of muscle RC deficiency, the *Cox10* homozygous knockout mouse 80. Whether ROS play a role in the induction of the Akt1–FGF21 signalling pathway in RC-deficient muscle fibres and whether the activation of this pathway contributes to the slow progression of RC deficiency have not been investigated.

Despite the fact that the mechanism of signal transduction remains unknown, it now seems clear that dysfunctional or damaged mitochondria can send a retrograde signal to the nucleus and further to other cell types, to promote an adaptive defence response. According to the mitohormesis theory and to the gradual ROS response hypothesis, ROS are mediators of the stress responses in general and of the stress response to age-dependent damage in particular. However, to date, the exact mechanism whereby the ROS-independent age-dependent damage could impact on mitochondrial function has not been clarified.

### The gradual ROS response theory of ageing: impact on therapeutic strategies for the treatment of age-related human diseases

Mitochondrial ROS are involved in a wide range of age-related diseases such as diabetes, cancer and neurodegeneration. The mitochondrial free radical theory of ageing has logically led to intervention studies with the aim of decreasing the level of ROS for health benefits in patients with such disorders. However, a large number of clinical trials have failed to demonstrate beneficial effects in these pathologies of intervention with ROS-scavenging drugs 83. Given the complex roles of ROS described above, that is, to create damage and to elicit signalling, it is likely that increasing antioxidant capacity within an organism would limit not only oxidative damage but also the normal adaptation to physiological stress so that ROS scavenging would be a risky therapeutic strategy.

Cancer provides a good example of a common condition in which the role of ROS is complex and the results of clinical trials of agents to decrease oxidative stress show disparate results. Although it is not an universal feature of all human cancers, the Warburg effect is a well-characterized metabolic phenotype of many tumours. This effect consists of a shift from ATP generation by oxidative phosphorylation to ATP generation by glycolysis (reviewed in 84). It has been proposed that glycolytic metabolism arises as an adaptation to hypoxic conditions during the early avascular phase of tumour development. Although ATP production by glycolysis can be more rapid than ATP production by oxidative phosphorylation, it is also much less efficient in terms of the ratio of ATP generated to glucose consumed. This shift therefore demands that a very high rate of glucose uptake is implemented in tumour cells to meet the increased energetic demands of highly proliferating cells 84. In addition, it should be noted that tumour cells are also adapted to continuous production of biomass that requires metabolic reprogramming to create the many precursors needed to make membranes, proteins and nucleic acids. Consequently, the metabolic adaptation of a tumour cell extends beyond the Warburg effect, and the pentose phosphate pathway is also activated to allow the production of large quantities of reduced nicotinamide adenine dinucleotide phosphate (NADPH), an intermediate required for macromolecule biosynthesis 84. It is interesting that NADPH is also a crucial cofactor for the ROS defence system 85. Thus, inhibiting NADPH production by inhibition of the pentose phosphate pathway could represent an interesting therapeutic option as it would theoretically slow down macromolecule biosynthesis and also render the transformed cells more susceptible to oxidative damage 84. The generation of oxidative stress has also been shown to trigger apoptosis 86, 87, which could constitute an additional beneficial effect from a clinical perspective. Paradoxically, there is also evidence indicating that ROS, depending on their types and concentrations, can serve as signalling molecules in cell proliferation and survival 88–90. It is therefore very difficult to accurately predict the outcome of an intervention to decrease NADPH production *in vivo* and, more generally, the outcome of any intervention targeting ROS signalling. As an illustration of this complexity*,* in one clinical trial, it was found that antioxidant supplementation was associated with a reduction in cancer incidence in men after a follow-up of 7.5 years 91, but such antioxidant supplementation may only have beneficial effects on cancer incidence in healthy subjects with low baseline antioxidant levels 83, 92. In line with this suggestion, high-dose antioxidant supplementation may be deleterious after initiation of the initial phase of carcinogenesis and could therefore be ineffective in subjects with adequate antioxidant status 92. Consistent with this, a recent review of randomized trials comparing antioxidant supplementation versus placebo/no intervention on the incidence of gastrointestinal cancers reported no evidence for a preventive effect of antioxidant supplements 93 and in some cases antioxidant supplements even seemed to increase overall mortality.

Defective oxidative phosphorylation and oxidative stress have for a long time been amongst the most popular hypotheses to explain the pathogenesis of a variety of neurodegenerative disorders. A recent model has proposed that impaired oxidative phosphorylation is not a primary cause of late-onset neurodegeneration but rather secondary to other types of underlying mitochondrial problems 94 such as perturbation of mitochondrial dynamics, which impairs mitochondrial trafficking, interorganellar communication and mitochondrial quality control. Nevertheless, oxidative stress occurs at an early stage and is a well-established pathophysiological factor in neurodegenerative diseases such as Alzheimer's disease (AD) and could in principle constitute an interesting target for neuroprotective therapies. However, the use of antioxidants in the prevention or therapy of neurodegenerative disorders has provided conflicting results. A detailed review of the antioxidant clinical trials in AD and mild cognitive impairment 95, which is considered to be an early manifestation of AD, illustrates this clearly. For instance, a prospective study including 633 individuals, age 65 years or above, showed that 91 untreated participants were diagnosed with AD after 4.3 years of follow-up, whereas none of the 27 who received vitamin E supplements and none of the 23 who received vitamin A supplements developed AD, thus arguing for a protective role of antioxidants against development of AD 96. By contrast, the results of a recent study of 57 patients demonstrated that vitamin E lowered oxidative stress in a subset of AD patients without preventing loss of cognition. Furthermore, the intervention had a detrimental effect on cognition in patients in whom oxidative stress was not decreased 97. The list of such inconsistent results is very long and also applies to other neurodegenerative pathologies such as amyotrophic lateral sclerosis 98–103 and Parkinson's disease 104. Some of the discrepancies have been explained by differences in the disease stage in which the treatments were initiated, the bioavailability and dose of the antioxidants used or the pre-existing antioxidant status of the patients 83, 95. However, another plausible explanation, based on recent experimental results, may also reside in the multiple roles played by ROS in neural cells. On the one hand, oxidative stress is considered to be of central importance in chronic neurodegeneration but, on the other hand, ROS are potent inducers of several cellular defence mechanisms, for example, the selective autophagic degradation of mitochondria, also termed mitophagy 94. Cells seem to be able to distinguish fully functional mitochondria from those that are defective (i.e. cells that exhibit low membrane potential and elevated ROS), and the accumulation of defective mitochondria is thought to be prevented via a dynamic equilibrium of mitochondrial fusion and fission events, which equalizes the gene product content of the individual mitochondria in a cell 94. If continuous fusion and fission events are not sufficient to maintain mitochondrial quality, then selective mitophagy may be important 105, 106. At low levels, ROS have been reported to induce mitophagy 94, 105, 107, 108 and administration of antioxidant drugs may therefore not retard chronic degeneration but rather create adverse effects, as observed in some clinical trials. Results from interventions in humans together with the experimentally supported gradual ROS response theory point towards the need to clarify dose–response relationships and to identify therapeutic windows in which antioxidant supplementation may be a beneficial way to treat oxidative damage without abolishing the beneficial effects of ROS signalling.

## Conclusion

Due to progress during the last decade in the understanding of mtDNA mutations, ROS and ageing, it is now clear that the mitochondrial free radical theory of ageing may be in a moribund stage, at least in its original ROS-dependent form. This theory initially provided a very interesting framework and led to many crucial discoveries in the field of mitochondrial function and ageing. However, accumulation of evidence that is not reconcilable with the theory has led to the necessity to formulate new concepts that encompass a wider range of experimental results. Amongst these new emerging theories is the gradual ROS response hypothesis 7 proposing that ROS represent a stress signal in response to age-dependent damage and thus are a consequence rather than a cause of ageing. This modified theory provides a new way of interpreting experimental results that were previously conceptually conflicting with the mitochondrial free radical theory of ageing. The development of specific tools that would allow a more accurate determination of ROS levels on the one hand and of oxidative damage on the other seems to be essential for future progress in this area. Additional experiments need to be conducted in animal models to further unravel the mechanisms linking ROS to mtDNA mutations and ageing. First, as mentioned previously, it would be helpful to determine whether a reduction in somatic levels of mtDNA mutations will prolong lifespan in experimental models. Secondly, the signalling pathway that mediates the ROS-mediated stress response, as proposed by Hekimi and colleagues, needs to be further characterized. ROS are widely implicated in different types of human diseases and the design of intervention studies with the aim of treating human oxidative stress-mediated age-related diseases will depend on experimental progress to better understand how ROS can be both beneficial and damaging.

## Abbreviations

AD: Alzheimer's disease
BER: base excision repair
DR: dietary restriction
GDH: glutamate dehydrogenase
GPx: glutathione peroxidase
IDH: isocitrate dehydrogenase
mtDNA: mitochondrial DNA
RC: respiratory chain
ROS: reactive oxygen species
SOD: superoxide dismutase
8-oxoG: 8-oxo-2′deoxyguanosine
